# A systematic review and meta-analysis on the radiation dose of computed tomography in hybrid nuclear medicine imaging

**DOI:** 10.1186/s40658-023-00553-8

**Published:** 2023-05-25

**Authors:** Gwenny Verfaillie, Caro Franck, An De Crop, Laurence Beels, Yves D’Asseler, Klaus Bacher

**Affiliations:** 1 Department of Human Structure and Repair, Ghent University, Ghent, Belgium; 2 mVISION, Faculty of Medicine and Health Sciences, Antwerp University, Antwerp, Belgium; 3 Department of Nuclear Medicine, AZ Delta, Roeselare, Belgium; 4 Department of Radiology, AZ Delta, Roeselare, Belgium; 5 Department of Nuclear Medicine, AZ Groeninge, Kortrijk, Belgium; 6 Department of Nuclear Medicine, Ghent University Hospital, Ghent, Belgium; 7 Department of Diagnostic Sciences, Ghent University, Ghent, Belgium

**Keywords:** Nuclear medicine, Hybrid imaging, DRLs, CTDI, DLP

## Abstract

**Background:**

While diagnostic reference levels (DRLs) are well-established for the radiopharmaceutical part, published DRLs for the CT component of positron emission tomography/computed tomography (PET/CT) and single photon emission computed tomography/computed tomography (SPECT/CT) are limited. This systematic review and meta-analysis provides an overview of the different objectives of CT in hybrid imaging and summarizes reported CT dose values for the most common PET/CT and SPECT/CT examinations. Also, an overview of already proposed national DRLs is given.

**Methods:**

A systematic literature search was performed to identify original articles reporting CT dose index volume (CTDI_vol_), dose-length product (DLP) and/or national DRLs for the most frequently performed PET/CT and/or SPECT/CT examinations. Data were grouped according to the clinical objective: diagnostic (D-CT), anatomical localisation (AL-CT) or attenuation correction (AC-CT) CT. Random-effects meta-analyses were conducted.

**Results:**

Twenty-seven articles were identified of which twelve reported national DRLs. For brain and tumour PET/CT imaging, CTDI_vol_ and DLP values were higher for a D-CT (brain: 26.7 mGy, 483 mGy cm; tumour: 8.8 mGy, 697 mGy cm) than for an AC/AL-CT (brain: 11.3 mGy, 216 mGy cm; tumour: 4.3 mGy, 419 mGy cm). Similar conclusions were found for bone and parathyroid SPECT/CT studies: D-CT (bone: 6.5 mGy, 339 mGy cm; parathyroid: 15.1 mGy, 347 mGy cm) results in higher doses than AL-CT (bone: 3.8 mGy, 156 mGy cm; parathyroid: 4.9 mGy, 166 mGy cm). For cardiac (AC-CT), mIBG/octreotide, thyroid and post-thyroid ablation (AC/AL-CT) SPECT/CT pooled mean CTDI_vol_ (DLP) values were 1.8 mGy (33 mGy cm), 4.6 mGy (208 mGy cm), 3.1 mGy (105 mGy cm) and 4.6 mGy (145 mGy cm), respectively. For all examinations, high variability in nuclear medicine practice was observed.

**Conclusion:**

The large variation in CT dose values and national DRLs highlights the need for optimisation in hybrid imaging and justifies the clinical implementation for nuclear medicine specific DRLs.

**Supplementary Information:**

The online version contains supplementary material available at 10.1186/s40658-023-00553-8.

## Introduction

In nuclear medicine, positron emission tomography (PET) and single photon emission computed tomography (SPECT) imaging often lack morphological information needed to localise the disease [1]. The combination of PET or SPECT with x-ray computed tomography (CT) provides essential anatomical information, thereby improving the quality of and the confidence in the nuclear medicine diagnosis [2]. Another advantage of the CT component in hybrid imaging is that it can be used for attenuation correction of the functional images.

Today, hybrid imaging modalities are well-established tools in nuclear medicine departments and play a vital role in the daily workflow of clinicians [3, 4]. The risks associated with the radiation dose from PET/CT and SPECT/CT are generally far outweighed by the benefits of the procedure when used appropriately. However, dual-modality imaging results in increased radiation exposures due to the combined dose from the CT component and the radiopharmaceutical. In nuclear medicine, CT acquisitions may be performed for different reasons. Depending on the clinical task and the image quality requirements, the radiation dose to the patient may differ. For attenuation correction (AC) and anatomical localisation (AL) of the emission data the CT dose can be relatively small while for diagnostic (D) purposes higher exposure levels are required. In addition, multimodality examinations are often used to monitor treatment response which require multiple examinations. It is thus important to be aware of the additional dose to the patient from the CT component of the scan. Several studies have reported comparable or higher effective doses resulting from the CT component of an ^18^F-fluorodeoxyglucose (^18^F-FDG) whole body PET/CT examination, compared to the dose from the radiopharmaceutical. [5–9] Depending on the calculation method, applied conversion coefficients and scan protocol, reported effective doses resulting from the CT component may range from 1 to around 16 mSv [3, 6, 8, 10–12].

Most hybrid imaging systems using a separate, conventional CT system often have separate protocols for diagnostic-, localisation- and/or attenuation correction-exclusive purposes. Also, hybrid imaging devices exist that are only able to perform localisation and/or attenuation correction CT scans. They are called low-dose CT devices. To compare the dose levels of different national and international nuclear medicine departments, CT dose index (CTDI_vol_) is the most relevant metric [13]. Dose-length product (DLP) on the other hand depends on the CTDI_vol_ as well as on the scan length applied at individual centres. These CT dose metrics are determined as standard to a 16 or 32 cm diameter IEC CT dosimetry phantom, also called CTDI phantom. In general, dose indicators for body examinations are reported to the 32 cm phantom while for head examinations they are reported to the 16 cm phantom. However, some examinations, e.g. those of the neck region including thyroid and cervical spine, may be reported either to a 16 or 32 cm phantom. It is therefore important to take this into account when comparing dose levels.

Unlike in diagnostic radiology, published dose reference levels (DRLs) for CT used in hybrid imaging are limited. The need to optimise the use of CT in hybrid imaging is high as little standardisation in nuclear medicine practice exists. The objective of this systematic review and meta-analysis is to provide an overview on the clinical use of CT in nuclear medicine along with the corresponding CT doses and CT DRLs.

## Materials and methods

The Preferred Reporting Items for Systematic Reviews and Meta-analyses (PRISMA) statement [14] was used as a guidance to conduct this systematic review and meta-analysis.

### Search strategy

A systematic literature search of the electronic databases Embase, Medline (through the PubMed interface), Web of Science and Scopus was performed to find all essential studies published until March 2022. Therefore, the research question was translated into three key concepts: adults (population), PET/CT or SPECT/CT imaging (intervention) and CT radiation dose (outcome). Selected keywords, based on retrieved articles in the initial search and medical subject headings (Medline), Emtree (Embase) or Index (Scopus) Terms, were grouped according to the key concepts and combined with the appropriate Boolean operators. In order to avoid missing critical studies, keywords were chosen carefully to enable a wide sensitive search. Similar search queries were used for the different databases and no restrictions on language were applied. In addition, the grey literature source Google Scholar was reviewed as well. To identify any further potential studies, the reference lists of included articles were screened.

### Study selection

**Fig. 1:**
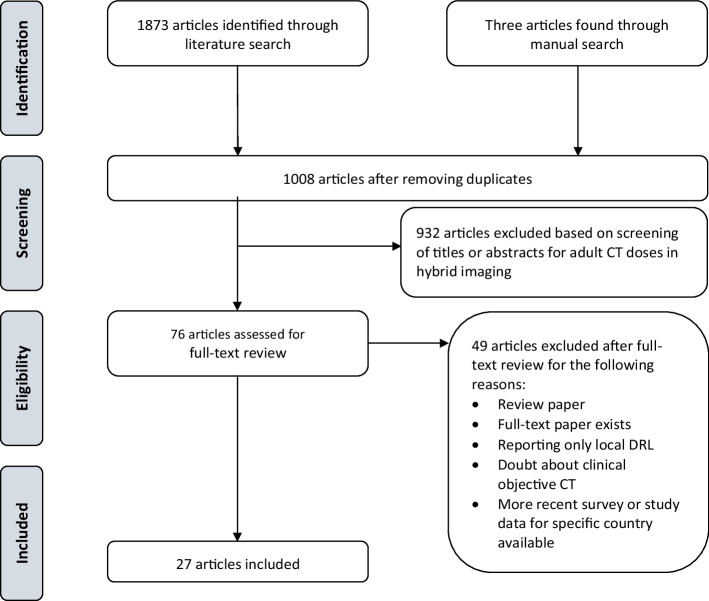
Flow diagram of included and excluded studies for the systematic review and meta-analysis

Initial screening of the literature was performed based on the titles and abstracts of all identified articles (Fig. 1). Only articles reporting CT dose index (CTDI_vol_), dose-length product (DLP) and/or diagnostic reference levels (DRLs) for adult PET/CT and/or SPECT/CT examinations were included. Articles and conference abstracts published in languages other than English, Dutch, French or German were excluded.

After a full text review, articles meeting one of the following exclusion criteria were rejected as well: (i) review paper, (ii) conference abstract of which a full-text paper exists, (iii) study reporting only local DRLs (insufficient dose data reported), (iv) doubt about clinical purpose CT acquisition (attenuation correction only, AC-CT, attenuation correction and localisation, AC/AL-CT, or diagnostic, D-CT), (v) survey or study from a country for which more recent data were available (Fig. 1).

### Data extraction

From the included articles, the following data were extracted: year of publication, country in which the study was performed, hybrid system type (PET/CT or SPECT/CT) and number of hybrid systems included in the study. For each hybrid imaging protocol the objective of the CT examination (AC-CT, AC/AL-CT or D-CT), mean CTDI_vol_ and DLP were extracted. Where possible, the used reference phantom size (16 or 32 cm diameter IEC CT dosimetry phantom) was extracted. If available, also reported national DRLs were gathered.

### Statistical analysis

To allow between-study variance, the random-effects model for meta-analyses (DerSimonian–Laird method) was used which attributes a weight, *W*_*jR*_*,* to each study *j* as follows:

$${W}_{jR}=\frac{1}{{\sigma }^{2}+{\tau }^{2}}$$

with *σ*^2^ the within-study variance, based on the standard errors (SEs) of the observed dose values, and the Dersimonian–Laird estimator *τ*^2^ (Tau-square) as estimator of the between-study variance [15]. Standard errors of the mean CTDI_vol_ and DLP values were calculated by dividing the mean standard deviation (SD) by the square root of the number of included hybrid imaging systems in the study ($\mathrm{SE}=\mathrm{SD}/\sqrt{n}$). Not all included articles reported means and/or standard deviations. In the absence of means and SDs, one or both values were calculated using the approach described by Hozo et al. [16]. When only the interquartile range was provided, the SD was estimated by dividing this range by 1.35 [17]. If no SD or range values were reported, a pooled SD was calculated from the mean SD values of the other studies included in the specific meta-analysis.

To analyse statistical heterogeneity the Cochran *Q* test, also known as the Chi-square test, and the *I*^2^ test, or Higgins *I*^2^ test, were performed. Whereas the Chi-square test only gives an indication whether or not heterogeneity is present, the *I*^2^ statistics describes the percentage of total variation across studies due to heterogeneity rather than chance [18]. *I*^2^ values of 25%, 50% and 75% are considered to reflect low, moderate and high heterogeneity, respectively [19]. To determine the effect of removing studies on the overall model, studies were excluded one at a time. Publication bias was evaluated based on the created funnel plots.

For each hybrid imaging examination, a separate random-effect meta-analysis was performed with CTDI_vol_ and DLP as primary outcome variables. First, random-effect meta-analyses were carried out for each examination depending on the clinical objective of the CT (attenuation correction only, attenuation correction and localisation or diagnostic). Secondly, random-effect meta-analyses were performed independent of the clinical objective of the CT equipment for each hybrid imaging examination if possible. For each meta-analysis, forest plots were created which present pooled estimated means, 95% confidence intervals (CI) and the *I*^2^ percentage.

All analyses were performed using the Cochrane statistical package RevMan 5.3 (Review Manager 5.3 [15]).

## Results

The combined search strategy identified 1873 articles: 642 from Embase, 499 from Medline, 330 from Web of Science, 399 from Scopus, 2 from Google Scholar and 1 from another source. After removing the duplicates, the titles and abstracts of the 1008 unique citations were screened. Of these, 76 articles met the criteria for a full-text review. Finally, 49 articles were excluded due to meeting one of the above-mentioned exclusion criteria. Eventually, 27 articles were included in this systematic review and meta-analysis (Fig. 1 and Table 1). Sixteen of these articles reported CT dose values resulting from a national survey [5, 8, 11, 13, 20–31].

**Table 1:** Overview of the included articles: author, year of publication, country in which the study was performed, number of included PET/CT and/or SPECT/CT devices in the study and whether nDRLs were reported

Author	Year	Country	N° of included PET/CT	N° of included SPECT/CT	Data on nDRLs
Tzampazidou et al*.* [32]	2021	Greece	1		No
Masoomi et al*.* [29]	2021	Kuwait	8		Yes
Peric et al*.* [31]	2021	Slovenia	3		No
Abe et al*.* [30]	2020	Japan	–	–	Yes
Alkhybari et al*.* [22]	2019	Australia, New Zealand	9 4		Yes
Bebbington et al*.* [24]	2019	Nordic countries: Denmark, Finland, Norway and Sweden	34	49	Yes
Chipiga et al*.* [25]	2019	Russia	26		No
Masoomi et al*.* [27]	2019	Kuwait	7		Yes
Brindhaban et al*.* [33]	2019	Kuwait		7	No
Lima et al*.* [13]	2018	Switzerland	19	24	Yes
Iball et al*.* [8]	2017	United Kingdom	33	43	Yes
ARPANSA [20]	2017	Australia	–	–	Yes
Avramova-Cholakova et al*.* [23]	2017	Bulgaria	6		No
Marti-Climent et al*.* [12]	2017	Spain	1		No
Tonkopi et al*.* [3]	2016	Canada		1	No
Kwon et al*.* [26]	2016	Korea	105		Yes
Sireus et al*.* [34]	2016	Italy	1		No
Abdollahi et al*.* [35]	2016	Iran		1	No
Jallow et al*.* [36]	2016	USA	158		Yes
Rausch et al*.* [37]	2016	Austria		1	No
Alessio et al*.* [21]	2015	USA	35		No
Avramova-Cholakova et al*.* [11]	2015	Bulgaria		4	Yes
Nye et al*.* [28]	2014	USA		35	No
Tonkopi et al*.* [9]	2013	Canada	1		No
Kaushik et al*.* [38]	2013	India	1		No
Etard et al*.* [5]	2012	France	56		Yes
Brix et al*.* [6]	2005	Germany	4		No

N°, number; nDRLs, national diagnostic reference levels; ARPANSA, Australian Radiation Protection and Nuclear Safety Agency

Only a limited number of studies have thus published CTDI_vol_ and DLP values resulting from the CT acquisition of dual-modality imaging protocols. The same applies to national diagnostic reference levels (nDRLs). Although DRLs are intended to promote harmonisation and good standards of practice such that radiation doses to patients undergoing clinical procedures involving ionising radiation are minimised as far as reasonably practicable, for the most part in nuclear medicine only DRLs for injected activities are currently available [13].

Figures S1–S8 (Additional file 1) and Tables 2 and 3 give an overview of, respectively, the reported CT radiation doses and proposed national DRLs for the most performed hybrid imaging procedures in different countries. The data reported from Australia, Bulgaria, France, Korea, Kuwait, New Zealand, Switzerland, the United Kingdom (UK), the United States of America (USA), the Nordic countries (including Denmark, Finland, Norway and Sweden) and recently also Japan are derived from nationwide surveys in which participants were asked to provide as much information as possible about CT protocol settings utilised, the examined patients, the radiation dose and the intended aim of the scan. The dose data of the Russian Federation are the result of a survey performed in twelve Russian regions. For the other reported countries, radiation doses were extracted from one up to seven hybrid imaging modalities mostly collected by university hospitals. Although some meta-analyses included only a few studies, no evidence of publication bias was observed on visual inspection of the funnel plots.

**Table 2:** Overview of published national diagnostic reference levels for CT in PET/CT

Modality	Examination	Country	CTDI_vol_ (mGy)	DLP (mGy cm)
PET/CT	^18^F-FDG whole body	Australia	4.41	474
		France	8.0	750
		Japan	6.1	600
		Korea	5.96	560
		Kuwait	4.1	684
		New Zealand	13.07	1319
		Switzerland	5.0	720
		USA	9.8	–
	^18^F-FDG half body	Kuwait	5.0	537
		Nordic countries	2.9	310
		Switzerland	6.0	620
		United Kingdom	4.3	400
	^18^F-FDG brain ﻿(AC/AL-CT)	Nordic countries	6.4	148
		Switzerland	7.0	100
	^18^F-FDG brain (D-CT)	Japan	31	640

AC/AL-CT, attenuation correction and anatomical localisation CT; D-CT, diagnostic CT

### CT doses in PET/CT

CT dose data of ^18^F-FDG PET/CT examinations were reported in 20 articles. Of these, 13 summarised the results of a national survey.

#### Meta-analysis

Figure S1 and Figure S2 (Additional file 1) display the mean CTDI_vol_ and DLP values reported in the literature for the frequently performed ^18^F-FDG PET/CT brain and tumour imaging examinations, respectively.

##### Brain imaging

Eight studies, related to eight different countries, contained CT dose data on ^18^F-FDG PET/CT brain examinations. Of these, six studies reported CTDI_vol_ and/or DLP values for CT examinations with as objective attenuation correction and localisation. For six countries, results were gathered as well for brain examinations with a diagnostic CT scan.

Without distinguishing the purpose of the CT scan, the overall pooled mean CTDI_vol_ was 15.2 mGy (95% CI 10.5–19.8; *I*^2^ = 62). For attenuation correction and localisation CT scans, a pooled mean CTDI_vol_ of 11.3 mGy (95% CI 7.9–14.7; *I*^2^ = 42) was found, while for diagnostic scans this value was 26.7 mGy (95% CI 14.5–38.8; *I*^2^ = 53) (Additional file 1: Fig. S1). The overall pooled mean DLP was 350 mGy cm (95% CI 234–465; *I*^2^ = 91): 216 mGy cm (95% CI 101–331; *I*^2^ = 77) when the CT scan was used for attenuation correction and localisation and 483 mGy cm (95% CI 325–640; *I*^2^ = 86) when the purpose of the scan was diagnostic (Additional file 1: Fig. S1).

##### Tumour imaging

Twenty-one articles, related to results for nineteen countries, included CT dose data on ^18^F-FDG PET/CT tumour imaging examinations. For seventeen countries, eighteen studies reported CTDI_vol_ and/or DLP values of examinations performed with an attenuation correction and localisation CT scan. Only eight studies reported CT dose data of PET/CT tumour imaging examinations executed with a diagnostic CT scan.

The overall pooled mean CTDI_vol_ was 5.6 mGy (95% CI 4.6–6.6; *I*^2^ = 94). When differentiating between the clinical CT objectives, a pooled mean of 4.3 mGy (95% CI 3.3–5.3; *I*^2^ = 94) and 8.8 mGy (95% CI 6.4–11.1; *I*^2^ = 83) was found for examinations using an attenuation correction and localisation CT scan and diagnostic CT scan, respectively (Additional file 1: Fig. S2). Meta-analysis of the mean DLP values resulted in an overall pooled mean DLP of 515 mGy cm (95% CI 430–560; *I*^2^ = 92). For the attenuation correction and localisation, CT scan the pooled mean DLP was 419 mGy cm (95% CI 335–503; *I*^2^ = 89), while for the diagnostic scan this was 697 mGy cm (95% CI 476–918; *I*^2^ = 94) (Additional file 1: Fig. S2).

#### Published national DRLs

From the sixteen surveys included in this review only ten reported suggested national DRLs for the CT component of the included PET/CT examinations. Table 3 gives an overview of the published proposed national DRLs (nDRLs).

**Table 3:** Overview of published national diagnostic reference levels for CT in SPECT/CT

Modality	Examination	Country	CTDI_vol_ (mGy)	DLP (mGy cm)
SPECT/CT	^99m^Tc bone imaging (AC/AL-CT)	Australia	–	240
		Bulgaria	3.0	200
		Nordic countries	4.0	215
		United Kingdom	4.9	150
	^99m^Tc bone pelvis ﻿(AC/AL-CT)	Switzerland	10.0	410
	^99m^Tc bone spine ﻿(AC/AL-CT)		5.0	190
	^99m^Tc bone extremities ﻿(AC/AL-CT)		17.0	380
	^99m^Tc bone imaging (D-CT)	Japan	5	380
	^99m^Tc cardiac imaging ﻿(AC-CT)	Australia	–	40
		Bulgaria	3	70
		Japan	4.1	85
		Nordic countries	2.2	53
		Switzerland	2.0	40
		United Kingdom	2.1	36
	^99m^Tc cardiac imaging ﻿(D-CT)	Japan	4.5	180
	mIBG/octreotide ﻿(AC/AL-CT)	Switzerland	5.0	250
		United Kingdom	5.5	240
	^99m^Tc parathyroid imaging (AC/AL-CT)	Australia	–	205
		Bulgaria	6.0	160
		Nordic countries	5.7	199
		Switzerland	4.0	160
		United Kingdom	5.6	170
	^131^I post-thyroid ablation (AC/AL-CT)	Bulgaria	4.0	160
		Switzerland	4.0	160
		United Kingdom	5.9	210
	^99m^Tc thyroid imaging (AC/AL-CT)	Bulgaria	4.0	170
		Switzerland	4.0	160

AC-CT, attenuation correction CT; AC/AL-CT, attenuation correction and anatomical localisation CT; D-CT, diagnostic CT

### CT doses in SPECT/CT

From the 27 included articles, 11 reported CT dose data of SPECT/CT examinations. For six countries, results were obtained from a national survey.

#### Meta-analysis

Figure S3 to S8 (Additional file 1) give an overview of the mean CTDI_vol_ and DLP values reported in literature for the most frequently performed SPECT/CT examinations.

##### Bone scan

Six articles reported CTDI_vol_ and/or DLP values of ^99m^Tc SPECT/CT bone scan examinations. All studies, except one, summarised data for examinations with an attenuation correction and localisation CT scan. Three studies included dose data when a diagnostic CT scan was used.

When the CT objective is not taken into account, the overall pooled mean CTDI_vol_ was 5.6 mGy (95% CI 2.5–8.7; *I*^2^ = 91). For attenuation correction and localisation CT scans, a pooled mean CTDI_vol_ of 3.8 mGy (95% CI 2.3–5.3; *I*^2^ = 52) was found, while for diagnostic scans this value was 6.5 mGy (95% CI −1.9–14.9; *I*^2^ = 92) (Additional file 1: Fig. S3). The overall pooled mean DLP was 224 mGy cm (95% CI 149–298; *I*^2^ = 64): 156 mGy cm (95% CI 124–189; *I*^2^ = 0) when the CT scan was used for attenuation correction and localisation and 339 mGy cm (95% CI 180–497; *I*^2^ = 51) when the purpose of the scan was diagnostic (Additional file 1: Fig. S3).

##### Cardiac imaging

Ten studies resulted into CTDI_vol_ and/or DLP values of ^99m^Tc SPECT/CT cardiac examinations with attenuation correction of the SPECT images as only reason for performing a CT scan.

A pooled mean of 1.8 mGy (95% CI 1.4–2.1; *I*^2^ = 13) and 33 mGy cm (95% CI 28–38; *I*^2^ = 44) was found for CTDI_vol_ and DLP, respectively (Additional file 1: Fig. S4).

##### mIBG/octreotide imaging

Only two articles published CTDI_vol_ and DLP data for mIBG/octreotide SPECT/CT studies. Here, the CT scan serves for attenuation correction and localisation purposes.

Meta-analysis resulted into a weighted pooled mean CTDI_vol_ of 4.6 mGy (95% CI 3.8–5.4; *I*^2^ = 0) and a weighted pooled mean DLP of 208 mGy cm (95% CI 168–248; *I*^2^ = 0) (Additional file 1: Fig. S5).

##### Thyroid imaging

Seven articles contained CT dose data on SPECT/CT thyroid imaging in general. Of these, six reported CTDI_vol_ and/or DLP values on ^99m^Tc parathyroid imaging with attenuation correction and localisation as CT objective. Three of them also included data when a diagnostic CT scan was performed instead. Only two of the seven articles contained CTDI_vol_ and DLP values of ^99m^Tc thyroid examinations. In this case, the CT scan is just used for attenuation correction and localisation. Three of the seven studies also reported attenuation correction and localisation CT dose data on ^131^I post-thyroid ablation examinations.

For parathyroid imaging, the overall pooled mean CTDI_vol_ was 6.2 mGy (95% CI 4.0–8.0; *I*^2^ = 73): 4.9 mGy (95% CI 4.1–5.7; *I*^2^ = 15) when the CT scan was used for attenuation correction and localisation and 15.1 mGy (95% CI 10.8–19.4; *I*^2^ = 0) when the purpose of the scan was diagnostic (Additional file 1: Fig. S6). Meta-analysis of the mean DLP values resulted in an overall pooled mean DLP of 194 mGy cm (95% CI 150–238; *I*^2^ = 63). For the attenuation correction and localisation, CT scan the pooled mean DLP was 166 mGy cm (95% CI 142–190; *I*^2^ = 0), while for the diagnostic scan this was 347 mGy cm (95% CI 211–484; *I*^2^ = 36) (Additional file 1: Fig. S6).

The pooled mean CTDI_vol_ and DLP for a SPECT/CT thyroid scan were 3.1 mGy (95% CI 0.8–5.3; *I*^2^ = 85) and 105 mGy cm (95% CI 53–157; *I*^2^ = 62), respectively (Additional file 1: Fig. S7).

For post-thyroid ablation examinations, a pooled mean CTDI_vol_ of 4.6 mGy (95% CI 3.6–5.4; *I*^2^ = 0) was found (Additional file 1: Fig. S8). The pooled mean DLP was 145 mGy cm (95% CI 96–193; *I*^2^ = 58) (Additional file 1: Fig. S8).

#### Published national DRLs

From the sixteen surveys included in this review only six reported suggested national DRLs for the CT component of the included SPECT/CT examinations. Table 3 gives an overview of the published proposed national DRLs (nDRLs).

## Discussion

The radiation dose delivered to the patient by hybrid imaging is increased as both the CT component and the nuclear medicine component (PET or SPECT) are high-dose examinations. The large variation in dose levels is due to different factors such as the clinical use of the CT data and the range of CT technologies that are used in hybrid imaging systems. Since CT images can be used for attenuation correction only, attenuation correction and anatomical localisation or diagnostic purposes, lower patient exposures are often sufficient to answer the relevant clinical question.

### CT doses in PET/CT

Because of the complex anatomy of the brain, precise localisation of suspicious foci of FDG uptake is difficult. Iball et al. (UK, [8]), Kaushik et al. (India, [38]) and Marti-Clement et al. (Spain, [12]) reported a CTDI_vol_ of around 13 mGy for a CT scan with as purpose attenuation correction and localisation, while a value of 7.1 mGy was found during a Swiss survey. A Nordic-wide survey, gathering data from facilities in Denmark, Finland, Norway, and Sweden, even resulted in a mean value of 15 mGy for the same examination [24]. As expected, higher doses were found for diagnostic CT scans, ranging from 16 to 46 mGy. The significantly wider range in diagnostic CTDI_vol_ values clearly comes forward in our meta-analysis suggesting that there is quite some room for further dose optimisation. It is important to notice that although CTDI_vol_ values of brain examinations should be linked to the 16 cm diameter reference phantom, they often are not. Therefore, CT dose values related to the 32 cm phantom should be converted to the 16 cm phantom when comparing CT doses. The majority of included studies reported the applied reference phantom and/or the conversion of CT doses to the 16 cm phantom. However, since not all studies reported this the observed range of CTDI_vol_ values may be influenced by values reported to a different reference phantom. Mean DLP values for an attenuation correction and anatomical localisation examination were smaller in the Swiss study compared to the UK, Spain, and Nordic study. This is mainly due to the much lower CTDI_vol_ while mean scan ranges are quite similar. DLP values for the diagnostic CTs, on the other hand, were comparable between the UK and Swiss study while a much higher value was reported in the Nordic study. However, the latter results from only one dataset and has to be taken with caution.

In this study, a distinction is made between whole body and half body examinations in which the body is scanned from head to mid-thigh and from neck to mid-thigh, respectively. In general, CT dose values are reported to the 32 cm reference phantom and scan ranges vary between 70 and 100 cm. The study of Alessio et al. (USA, [21]) had a significantly shorter scan range of 57 cm because the CT scans were performed from head to bladder. However, while the scan range was shorter, the mean CTDI_vol_ of a diagnostic CT scan was higher compared to other countries such as Switzerland (7 mGy) and the United Kingdom (5 mGy). As a result, higher than average DLP values were registered. A larger scan range (85 to 124 cm), nevertheless, causes the even higher DLP values found in the Nordic-wide survey [24]. For CT scans used for attenuation correction and localisation, most dose values were comparable with or higher than the values reported by Iball et al. [8], suggesting that there is still room for improvement in whole body PET/CT imaging. This improvement includes not only dose optimisation but also the description of a whole body CT scan. Most of the included studies described a whole body CT scan as a scan from head to mid-thigh while others called this a half body CT scan. Some studies also reported a second whole body scan defined as a scan from the head to the toes. This variability in description, and thus in scan length, is a confounding factor when analysing whole body CT dose data. Therefore, data of scans performed from head to toes were not used in this analysis.

Our meta-analyses demonstrated that the importance of the choice between a diagnostic or an attenuation correction and localisation CT scan should not be underestimated. Patients undergoing a diagnostic CT scan are exposed to significantly higher radiation doses. Depending on the nuclear medicine department, a diagnostic CT scan may also be acquired differently. In most cases they are taken with injected contrast medium and in breath-hold, as would be the case at the radiology department. However, a diagnostic CT as part of a nuclear medicine examination may be more justified if it reduces the need for the patient to attend a separate diagnostic CT on a dedicated diagnostic CT scanner for the same clinical indication.

Proposed national DRLs (nDRLs) for CTDI_vol_ of brain PET/CT examinations are quite similar for attenuation correction and localisation CT scans (Table 2). However, only a few countries reported nDRLs values. For the whole body PET/CT examinations some variance in proposed nDRLs is observed. Although some countries used a different approach to obtain nDRLs, calculating the 75th percentile of the distribution of all data instead of the 75th percentile of the distribution of mean dose values as proposed by the ICRP, this is probably not the cause for the observed variability. It is probably due to differences in the description and classification into clinical purpose of these examinations. Generic descriptions used to describe some CT procedures can thus be a potential source of ambiguity since a protocol with the same name can be applied to different body regions [8, 13]. The observed variability in both mean dose values and nDRLs suggests the need of CT dose optimisation together with a breakdown into the different descriptions of whole body examinations. Due to the lack of data needed to calculate the standard errors, no pooled estimate could be made for the nDRLs.

### CT doses in SPECT/CT

Screening for bone metastasis is a common indication for skeletal scintigraphy or bone imaging and therefore ^99m^Tc-diphosphonate is used. Because many benign skeletal processes demonstrate an increased radionuclide uptake, anatomic CT imaging can help differentiate benign from malignant lesions. ^99m^Tc-diphosphonate also plays a role in detecting or excluding the presence of infections. Localising the site of infection to soft tissue or bone with the help of CT data has a crucial impact on the type of therapy. As expected, dose values are higher for CT acquisitions with a diagnostic purpose. The Nordic study, however, reports a lower CTDI_vol_ for the diagnostic CT scan than for the localisation CT scan. However, because only one dataset reported CT doses of a diagnostic CT scan compared to 30 datasets in case of a localisation CT this result has to be taken with caution. It only suggests that a diagnostic CT as part of SPECT/CT bone examinations is not common in the Nordics [24]. In general, our meta-analyses suggest that mean CT doses are almost twice as high when the patient undergoes a diagnostic CT scan compared to an attenuation correction and localisation CT. However, since only three studies with a diagnostic CT scan are included of which one resulted in much lower dose values because of the above mentioned reason, mean diagnostic CT doses may be higher than observed in this meta-analysis. The lower observed dose values for the Nordic and Japanese study may also be explained by the use of a dedicated low-dose diagnostic CT protocol instead of a default diagnostic CT protocol. For the attenuation correction and localisation case, small pooled 95% confidence intervals are observed indicating that there already exists some consistency in SPECT/CT bone scan examinations between different countries.

SPECT cardiac imaging using ^99m^Tc is a sensitive modality to assess many cardiac diseases such as ischemia and infarctions but is affected by the attenuation of other organs in the field of view, including the breasts and diaphragm. Applying CT attenuation correction overcomes this issue to a large extent and offers high quality, artefact-free radionuclide images. In general, the CT component of a SPECT/CT cardiac examination is only used for attenuation correction. Nevertheless, a large variation in DLP values is found in literature with reported doses in Switzerland [13] being 10 times larger than in Canada [3]. CTDI_vol_ values resulting from national surveys are quite similar, suggesting that variations in DLP are due to differences in scan length.

MIBG and octreotide are both used to localise neuroendocrine tumours. Although reported dose values of the attenuation correction and localisation CT scan are scarce, doses between different countries seem to be similar. This is also demonstrated in our meta-analysis where the pooled mean CT doses, taking into account the weight of the different studies, are similar to the country specific doses.

Precise localisation of the (para)thyroid adenoma, which can be acquired by a ^99m^Tc SPECT/CT scan, is crucial for the success of minimally invasive (para)thyroidectomy. For parathyroid imaging, DLP values are almost all similar in the case that the CT is used for localisation and CTDI_vol_ values range from 4.1 to 7.4 mGy, while for diagnostic purposes CT doses are higher to achieve the required image quality. This is also demonstrated by our meta-analysis. Larger variations in DLP are seen for thyroid imaging suggesting a variation in scan length because CTDI_vol_ values are comparable. Following ablative therapy, the standard current practice in patients with well-differentiated thyroid cancer after radioiodine therapy is ^131^I SPECT/CT imaging. This allows accurate staging of the disease and tailors the management for the patient appropriately. Here as well, the CT doses are comparable between examinations performed in Bulgaria and the United Kingdom. The mean DLP found in the Canadian study is twice as high, suggesting larger scan lengths. However, this value is the result of a study on one SPECT/CT system and has to be taken with caution.

The SPECT/CT nDRLs for CTDI_vol_ (Table 3) are all in reasonable agreement with each other, expect those of the bone imaging protocols in Switzerland which is due to the discrete categorizations of individual bone protocols according to the different anatomical structures commonly scanned [13]. For all examinations, high variability in nuclear medicine practice was observed. Because of missing data needed to calculate the standard errors, no pooled estimate could be made for the nDRLs.

### Dose optimisation

Hybrid imaging is the combination of two potentially high-dose investigations which justifies the need for dose optimisation research. For the nuclear medicine component, the most important factor determining the image quality is the administered activity which must respect any diagnostic reference level and is chosen based on several patient dependent clinical and technical aspects. To reduce the radiation dose to the patient many factors can be considered. Of course, the administered activity can be lowered to reduce the radiation dose. This depends not only on patient characteristics but also on the reconstruction algorithm used. Moreover, the use of radiopharmaceuticals with shorter physical and biological half-lives generally results in lower doses. Technological improvements, such as new high-sensitivity collimators and high-efficiency camera systems with more sensitive detectors, further induce dose optimisation possibilities. [39, 40]

CT dose optimisation in hybrid imaging depends first of all on the purpose of the CT acquisition. When recent diagnostic CT data are available and when follow-up studies are to be performed a low-dose CT is suggested. This is also the case for anatomical localisation or focal pathology characterisation. A diagnostic CT is only suggested when recent CT data are not available and when detailed anatomical information is needed. Because today most PET/CT and SPECT/CT systems have a CT part with full diagnostic image quality, a diagnostic CT as part of the nuclear medicine examination can avoid the need for the patient to attend a second imaging examination which reduces the radiation dose to the patient. In general, CT dose reduction principles in nuclear medicine are the same as in radiology. Optimising the CT scanning parameters such as tube potential, tube load, rotation time, beam width, pitch, reconstructed image thickness and applied reconstruction kernel is a first but necessary step. However, the acquisition for particular body regions is greatly influenced by the patient size, requiring patient specific settings. Using automatic tube current modulation further reduces the radiation dose by adapting the tube current to the patient’s size and anatomy. Some systems also provide organ-based tube current modulation reducing the dose to radiosensitive organs close to the surface of the body. Also, the use of iterative reconstruction algorithms allows different dose savings. Moreover, recent developments in artificial intelligence technology have introduced deep learning-based reconstruction techniques [41, 42]. Preliminary phantom and clinical studies have shown the potential of deep learning reconstruction for further dose reduction [42]. Throughout this optimisation process, it is important to consider the image quality as well because it will be influenced by changes in the CT scan and reconstruction parameters. Together with the integrated dose reduction tools, the clinical task and corresponding image quality requirements will eventually impose practical limits on achievable CT dose reduction. Although almost never specifically mentioned, the CT technology and image quality requirements will have an impact on the observed CT doses. [39, 40]

### Limitations of the study

Although most CT protocols are determined as standard to the 16 and 32 cm IEC CT dosimetry phantom for head and body protocols, respectively, there may be some deviations. Therefore, it is important to convert the dose data, if necessary, to the dedicated reference phantom when comparing dose data between systems. However, most studies do not report the reference phantom in which the CTDI_vol_ and DLP values are defined which may be a confounding factor.

While the Chi-square test is statistically significant for almost all included PET/CT studies, clearly indicating the presence of heterogeneity, it is in half of the cases non-significant for the included SPECT/CT studies (Additional file 1). However, this does not mean that heterogeneity is completely absent. Because the Chi-square test has a low power when only a few studies are included, the *I*^2^ statistics is better suited to assess heterogeneity. Most study groups of the included PET/CT and SPECT/CT examinations show moderate or high statistical heterogeneity, as measured with the *I*^2^ statistic. This supports the use of random-effects meta-analyses. However, for some study groups low or no heterogeneity was found. Tests for heterogeneity tend to be underpowered for detecting small differences if the number of studies and their sample sizes are low. Nevertheless, it is better to have a biased and imprecise estimate than to have no estimate at all. Attention just needs to be paid as heterogeneity cannot necessarily be excluded [43]. Next to the number of included studies, heterogeneity can also be influenced by the number of included devices per study for each type of examination and the amount of patient data collected per device and examination type. A nationwide survey includes of course more data. Finally, heterogeneity can also be explained by the PET/CT or SPECT/CT equipment used. Older systems may not contain any or the same dose reduction tools as the newest devices have. Default scan protocols may vary from manufacturer to manufacturer and from model to model. Differences in operator’s preferences influence CT radiation doses too.

## Conclusion

The benefits of combining functional with anatomical information have increased the use of hybrid imaging modalities, such as PET/CT and SPECT/CT, in nuclear medicine. However, attention must be payed to the patient dose received by exposure to ionising radiation because both the CT and the nuclear medicine component are potentially high-dose examinations. While DRLs are well-established for the radiopharmaceutical part, this is not the case for the CT component. In this systematic review and meta-analysis, an overview was given of the published CT dose values for the most performed PET/CT and SPECT/CT examinations in- and outside Europe. The large variation in dose levels is due to different factors such as the clinical use of the CT, the used CT technology and the generic description of certain CT examinations. This justifies the requirement for DRLs specific to nuclear medicine practice. Some countries already proposed national DRLs for certain examinations. But here also, a wide variation in nuclear medicine practice was observed which highlights the need for optimisation in hybrid imaging.

## Supplementary Information

**Additional file 1.** Meta-analysis results for CTDI_vol_ and DLP of CT examinations in PET/CT and SPECT/CT.

## Data Availability

The authors declare that all data supporting the findings of this study are available within the article.

## Abbreviations

AC: Attenuation correction
AC-CT: Attenuation correction CT
AL: Anatomical localisation
AL-CT: Anatomical localisation CT
CI: Confidence interval
CT: Computed tomography
CTDI_vol_: CT dose index volume
D: Diagnostic
D-CT: Diagnostic CT
df: Degrees of freedom
DLP: Dose-length product
DRLs: Diagnostic reference levels
FDG: Fluorodeoxyglucose
mIBG: Meta-iodo-benzyl-guanidine
nDRLs: National diagnostic reference levels
PET: Positron emission tomography
PET/CT: Positron emission tomography/computed tomography
PRISMA: Preferred Reporting Items for Systematic Reviews and Meta-analyses
SD: Standard deviation
SE: Standard error
SPECT: Single photon emission computed tomography
SPECT/CT: Single photon emission computed tomography/computed tomography
