# Supplementary material for: A systematic review and meta-analysis on the radiation dose of computed tomography in hybrid nuclear medicine imaging
Source: EJNMMI Phys. 2023 May 25;10:32. doi: 10.1186/s40658-023-00553-8 (PMC10212852; doi:10.1186/s40658-023-00553-8)
Supplement: Supplementary file 1 — Additional file 1. Meta-analysis results for CTDIvol and DLP of CT examinations in PET/CT and SPECT/CT. [file 40658_2023_553_MOESM1_ESM.pdf]

SUPPLEMENTARY INFORMATION

A systematic review and meta-analysis on the radiation dose of Computed Tomography in hybrid nuclear medicine imaging

Gwenny Verfaillie[1], Caro Franck[2], An De Crop[3,4], Laurence Beels[5], Yves D'Asseler[6,7], Klaus Bacher[1]

[1] Ghent University, Department of Human Structure and Repair, Ghent, Belgium

[2] Antwerp University, mVISION, Faculty of Medicine and Health Sciences, Antwerp, Belgium

[3] AZ Delta, Department of Nuclear Medicine, Roeselare, Belgium

[4] AZ Delta, Department of Radiology, Roeselare, Belgium

[5] AZ Groeninge, Department of Nuclear Medicine, Kortrijk, Belgium

[6] Ghent University Hospital, Department of Nuclear Medicine, Ghent, Belgium

[7] Ghent University, Department of Diagnostic Sciences, Ghent, Belgium

This supplementary document presents the results of the meta-analyses for $CTDI_{vol}$ and DLP of CT examinations in PET/CT and SPECT/CT, including mean dose values, 95% confidence intervals, forest plots and results for statistical heterogeneity.

META-ANALYSIS RESULTS FOR CTDI$_{VOL}$ AND DLP OF CT EXAMINATIONS IN PET/CT

Brain imaging: AC/AL-CT

		CTDI$_{vol}$ (mGy)			DLP (mGy.cm)		
**Country**	**n**	**mean**	**95% CI**	**weight**	**mean**	**95% CI**	**weight**
Nordic countries	9	15.0	[2.1, 27.9]	6.2%	302	[77, 527]	13.7%
Spain	1	13.1	[11.1, 15.1]	46.2%	302	[243, 361]	27.0%
Switzerland	8	7.1	[3.0, 11.2]	30.5%	97	[32, 162]	26.6%
United Kingdom	7	12.6	[5.3, 19.9]	15.6%	236	[97, 375]	20.4%
Australia	-	-	-	-	125	[-238, 488]	7.5%
India	1	13.1	[-13.7, 39.9]	1.6%	197	[-277, 671]	4.9%
**Pooled estimate**		**11.3**	**[7.9, 14.7]**	**100%**	**216**	**[101, 331]**	**100%**

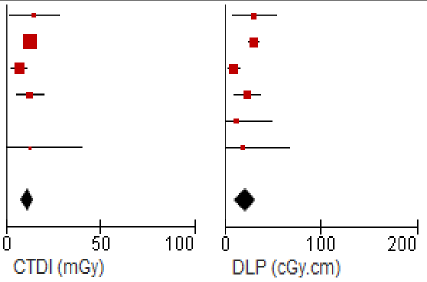

CTDI$_{vol}$:
Heterogeneity: $Tau^2 = 5.51$; $Chi^2 = 6.94$, $df = 4$ ($P = 0.14$); $I^2 = 42\%$
Test for overall effect: $Z = 6.52$ ($P < 0.00001$)
DLP:
Heterogeneity: $Tau^2 = 11822.36$; $Chi^2 = 22.09$, $df = 5$ ($P = 0.0005$); $I^2 = 77\%$
Test for overall effect: $Z = 3.68$ ($P = 0.0002$)

Brain imaging: D-CT

		CTDI$_{vol}$ (mGy)			DLP (mGy.cm)		
**Country**	**n**	**mean**	**95% CI**	**weight**	**mean**	**95% CI**	**weight**
Nordic countries	1	45.8	[22.5, 69.1]	16.3%	1068	[847, 1290]	16.1%
Russia	26	-	-	-	440	[397, 483]	23.2%
Switzerland	3	36.2	[19.0, 53.4]	22.4%	389	[283, 495]	21.3%
United Kingdom	3	16.4	[8.3, 24.5]	34.5%	315	[168, 462]	19.6%
India	1	20.6	[-2.7, 43.9]	16.3%	309	[88, 531]	16.1%
Japan	-	19.6	[-12.8, 52.0]	10.4%	384	[-360,1129]	3.7%
**Pooled estimate**		**26.7**	**[14.5, 38.8]**	**100%**	**483**	**[325, 640]**	**100%**

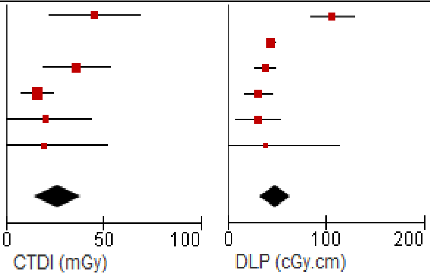

CTDI$_{vol}$:
Heterogeneity: $Tau^2 = 93.82$; $Chi^2 = 8.48$, $df = 4$ ($P = 0.08$); $I^2 = 53\%$
Test for overall effect: $Z = 4.30$ ($P < 0.0001$)
DLP:
Heterogeneity: $Tau^2 = 27133.92$; $Chi^2 = 35.92$, $df = 5$ ($P < 0.00001$); $I^2 = 86\%$
Test for overall effect: $Z = 6.02$ ($P < 0.00001$)

Fig. S1 Forest plot of reported mean CTDI$_{vol}$ and DLP values for ^{18}F-FDG PET/CT brain imaging divided by studies using an attenuation correction and anatomical localisation CT (AC/AL-CT) and a diagnostic CT (D-CT) (n, number of included PET/CT systems; CI, confidence interval; df, degrees of freedom; red square, size proportional to the weight of the individual study in the meta-analysis; black diamond, pooled estimate with length depicting the 95% CI)

Tumour imaging: AC/AL-CT

Country	n	CTDI$_{vol}$ (mGy) mean	CTDI$_{vol}$ (mGy) 95% CI	CTDI$_{vol}$ (mGy) weight	DLP (mGy.cm) mean	DLP (mGy.cm) 95% CI	DLP (mGy.cm) weight
Bulgaria	6	4.0	[2.5, 5.5]	7.0%	392	[225, 559]	7.8%
France	56	6.6	[6.2, 7.0]	8.3%	628	[575, 681]	10.8%
Germany	4	2.0	[1.1, 2.9]	7.8%	-	-	-
Greece	1	5.5	[0.8, 10.2]	2.8%	484	[67, 899]	3.0%
Italy	1	2.1	[-3.9, 8.1]	2.0%	-	-	-
Nordic countries	15	2.7	[2.2, 3.2]	8.3%	270	[221, 319]	10.9%
Slovenia	3	3.5	[2.2, 4.8]	7.4%	389	[263, 515]	9.0%
Spain	1	6.3	[2.6, 10.0]	3.8%	557	[149, 965]	3.1%
Switzerland	12	4.4	[2.7, 6.1]	6.7%	416	[267, 565]	8.3%
United Kingdom	28	3.4	[2.9, 3.9]	8.3%	326	[279, 373]	10.9%
Australia	8	3.3	[1.9, 4.7]	7.2%	350	[211, 489]	8.6%
Canada	1	4.3	[1.2, 7.4]	4.5%	368	[84, 652]	4.9%
India	1	4.3	[-1.7, 10.3]	2.0%	434	[32, 836]	3.1%
Korea	105	4.6	[4.1, 5.1]	8.3%	429	[386, 472]	11.0%
Kuwait	7	4.6	[3.5, 5.7]	7.7%	528	[393, 663]	8.7%
USA	158	7.0	[6.4, 7.7]	8.1%	-	-	-
**Pooled estimate**		**4.3**	**[3.3, 5.3]**	**100%**	**419**	**[335, 503]**	**100%**

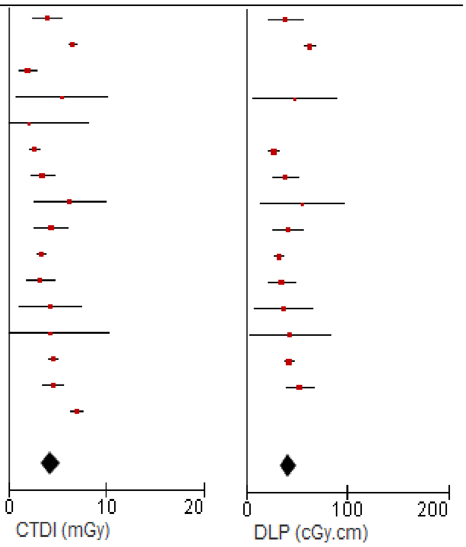

CTDI$_{vol}$:
Heterogeneity: $Tau^2 = 2.92$; $Chi^2 = 262.52$, df = 15 ($P < 0.00001$); $I^2 = 94\%$
Test for overall effect: $Z = 8.66$ ($P < 0.00001$)
DLP:
Heterogeneity: $Tau^2 = 16095.72$; $Chi^2 = 114.00$, df = 12 ($P < 0.00001$); $I^2 = 89\%$
Test for overall effect: $Z = 9.83$ ($P < 0.00001$)

Tumour imaging: D-CT

Country	n	CTDI$_{vol}$ (mGy) mean	CTDI$_{vol}$ (mGy) 95% CI	CTDI$_{vol}$ (mGy) weight	DLP (mGy.cm) mean	DLP (mGy.cm) 95% CI	DLP (mGy.cm) weight
Germany	4	11.7	[9.8, 13.6]	15.9%	-	-	-
Nordic countries	11	9.9	[8.4, 11.4]	16.5%	963	[826, 1100]	14.8%
Russia	26	-	-	-	890	[767, 1014]	15.0%
Switzerland	6	7.0	[4.5, 9.5]	14.7%	583	[393, 773]	14.1%
United Kingdom	6	5.0	[3.3, 6.7]	16.2%	363	[302, 424]	15.5%
India	1	6.6	[-5.3, 18.5]	3.1%	674	[45, 1303]	6.9%
Japan	-	4.2	[-1.0, 9.5]	9.1%	451	[-7, 910]	9.4%
New Zealand	4	11.6	[6.8, 16.4]	9.9%	992	[533, 1451]	9.4%
USA	35	11.7	[9.2, 14.2]	14.6%	672	[552, 792]	15.0%
**Pooled estimate**		**8.8**	**[6.4, 11.1]**	**100%**	**722**	**[488, 957]**	**100%**

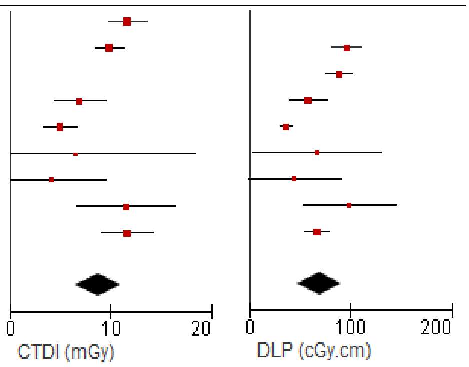

CTDI$_{vol}$:
Heterogeneity: $Tau^2 = 7.72$; $Chi^2 = 42.34$, df = 7 ($P < 0.00001$); $I^2 = 83\%$
Test for overall effect: $Z = 7.50$ ($P < 0.00001$)
DLP:
Heterogeneity: $Tau^2 = 80624.31$; $Chi^2 = 108.15$, df = 7 ($P < 0.00001$); $I^2 = 94\%$
Test for overall effect: $Z = 6.19$ ($P < 0.00001$)

Fig. S2 Forest plot of reported mean CTDI$_{vol}$ and DLP values for ^{18}F-FDG PET/CT tumour imaging divided by studies using an attenuation correction and anatomical localisation CT (AC/AL-CT) and a diagnostic CT (D-CT) (n, number of included PET/CT systems; CI, confidence interval; df, degrees of freedom; red square, size proportional to the weight of the individual study in the meta-analysis; black diamond, pooled estimate with length depicting the 95% CI)

META-ANALYSIS RESULTS FOR CTDI$_{VOL}$ AND DLP OF CT EXAMINATIONS IN SPECT/CT

Bone scan (^{99m}Tc): AC/AL-CT

		CTDI$_{vol}$ (mGy)			DLP (mGy.cm)		
**Country**	**n**	**mean**	**95% CI**	**weight**	**mean**	**95% CI**	**weight**
Bulgaria	4	5.7	[-0.6, 12.0]	5.0%	236.0	[-7, 479]	1.8%
Nordic countries	30	2.8	[2.1, 3.5]	51.9%	153.0	[114, 192]	68.4%
United Kingdom	32	4.5	[3.0, 6.0]	37.0%	150.0	[85, 215]	25.1%
Australia	-	-	-	-	170.0	[-12, 352]	3.2%
Canada	1	6.5	[0.8, 12.2]	6.0%	281.0	[22, 540]	1.6%
**Pooled estimate**		**3.8**	**[2.3, 5.3]**	**100%**	**156**	**[124, 189]**	**100%**

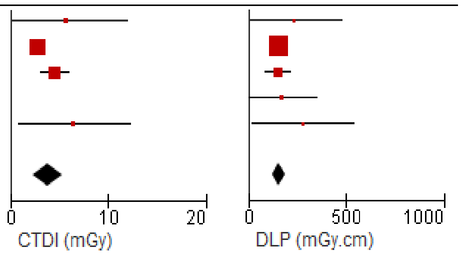

CTDI$_{vol}$:
Heterogeneity: $Tau^2 = 0.97$; $Chi^2 = 6.19$, $df = 3$ ($P = 0.10$); $I^2 = 52\%$
Test for overall effect: $Z = 5.04$ ($P < 0.00001$)
DLP:
Heterogeneity: $Tau^2 = 0.00$; $Chi^2 = 1.39$, $df = 4$ ($P = 0.85$); $I^2 = 0\%$
Test for overall effect: $Z = 9.45$ ($P < 0.00001$)

Bone scan (^{99m}Tc): D-CT

		CTDI$_{vol}$ (mGy)			DLP (mGy.cm)		
**Country**	**n**	**mean**	**95% CI**	**weight**	**mean**	**95% CI**	**weight**
Nordic countries	1	2.3	[-2.1, 6.7]	33.5%	255	[120, 390]	46.8%
United Kingdom	3	13.5	[11.0, 16.1]	35.7%	444	[309, 579]	46.8%
Japan	-	2.9	[-3.2, 9.1]	30.8%	180	[-413, 774]	6.4%
**Pooled estimate**		**6.5**	**[-1.9, 14.9]**	**100%**	**339**	**[180, 497]**	**100%**

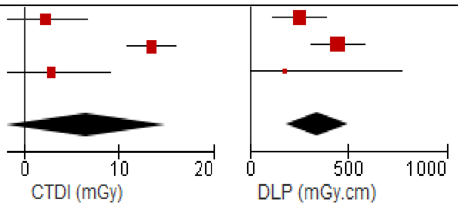

CTDI$_{vol}$:
Heterogeneity: $Tau^2 = 49.89$; $Chi^2 = 23.98$, $df = 2$ ($P < 0.00001$); $I^2 = 92\%$
Test for overall effect: $Z = 1.51$ ($P = 0.13$)
DLP:
Heterogeneity: $Tau^2 = 9100.71$; $Chi^2 = 4.06$, $df = 1$ ($P = 0.13$); $I^2 = 51\%$
Test for overall effect: $Z = 4.20$ ($P < 0.0001$)

Fig. S3 Forest plot of reported mean CTDI$_{vol}$ and DLP values for ^{99m}Tc SPECT/CT bone scan divided by studies using an attenuation correction and anatomical localisation CT (AC/AL-CT) and a diagnostic CT (D-CT) (n, number of included SPECT/CT systems; CI, confidence interval; df, degrees of freedom; red square, size proportional to the weight of the individual study in the meta-analysis; black diamond, pooled estimate with length depicting the 95% CI)

Cardiac (^{99m}Tc): AC-CT

Country	n	CTDI$_{vol}$ (mGy) mean	CTDI$_{vol}$ (mGy) 95% CI	CTDI$_{vol}$ (mGy) weight	DLP (mGy.cm) mean	DLP (mGy.cm) 95% CI	DLP (mGy.cm) weight
Austria	1	1.3	[-2.5, 5.1]	0.8%	35	[31, 39]	32.8%
Bulgaria	4	1.5	[-0.4, 3.4]	3.0%	27	[23, 31]	32.8%
Nordic countries	30	2.1	[1.4, 2.8]	17.1%	40	[30, 50]	15.5%
Switzerland	11	2.2	[0.8, 3.6]	5.8%	75	[-15, 165]	0.3%
United Kingdom	30	2.0	[1.3, 2.7]	17.1%	33	[23, 43]	15.5%
Australia	-	-	-	-	40	[11, 69]	2.6%
Canada	1	0.5	[-3.3, 4.3]	0.8%	7	[-117, 131]	0.2%
Iran	1	1.3	[0.9, 1.7]	41.4%	-	-	-
Japan	-	1.6	[-5.5, 8.7]	0.2%	45	[-70, 161]	0.2%
Kuwait	7	2.3	[1.5, 3.1]	13.9%	-	-	-
**Pooled estimate**		**1.8**	**[1.4, 2.1]**	**100%**	**33**	**[28, 38]**	**100%**

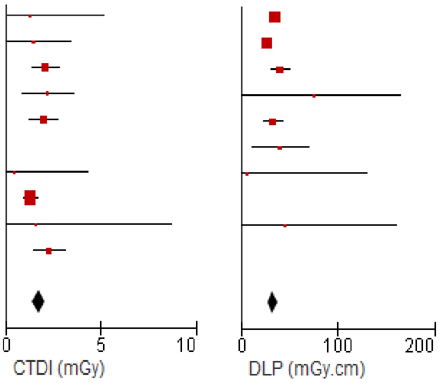

CTDI$_{vol}$:
Heterogeneity: Tau² = 0.04; Chi² = 9.19, df = 8 (P = 0.33); I² = 13%
Test for overall effect: Z = 10.19 (P < 0.00001)
DLP:
Heterogeneity: Tau² = 14.88; Chi² = 12.45, df = 7 (P = 0.09); I² = 44%
Test for overall effect: Z = 13.28 (P < 0.00001)

Fig. S4 Forest plot of reported mean CTDI$_{vol}$ and DLP values for ^{99m}Tc SPECT/CT cardiac imaging using an attenuation correction CT (AC-CT) (n, number of included SPECT/CT systems; CI, confidence interval; df, degrees of freedom; red square, size proportional to the weight of the individual study in the meta-analysis; black diamond, pooled estimate with length depicting the 95% CI)

mIBG/octreotide: AC/AL-CT

Country	n	CTDI$_{vol}$ (mGy) mean	CTDI$_{vol}$ (mGy) 95% CI	CTDI$_{vol}$ (mGy) weight	DLP (mGy.cm) mean	DLP (mGy.cm) 95% CI	DLP (mGy.cm) weight
Switzerland	6	4.6	[3.7, 5.5]	81.0%	210	[165, 255]	79.3%
United Kingdom	35	4.5	[2.7, 6.3]	19.0%	201	[113, 289]	20.7%
**Pooled estimate**		**4.6**	**[3.8, 5.4]**	**100%**	**208**	**[168, 248]**	**100%**

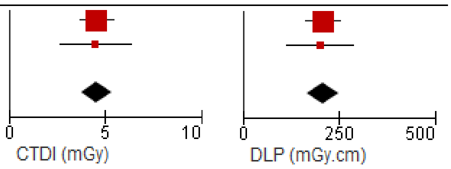

CTDI$_{vol}$:
Heterogeneity: Tau² = 0.00; Chi² = 0.01, df = 1 (P = 0.92); I² = 0%
Test for overall effect: Z = 11.31 (P < 0.00001)
DLP:
Heterogeneity: Tau² = 0.00; Chi² = 0.03, df = 1 (P = 0.86); I² = 0%
Test for overall effect: Z = 10.16 (P < 0.00001)

Fig. S5 Forest plot of reported mean CTDI$_{vol}$ and DLP values for SPECT/CT mIBG/octreotide imaging using an attenuation correction and anatomical localisation CT (AC/AL-CT) (n, number of included SPECT/CT systems; CI, confidence interval; df, degrees of freedom; red square, size proportional to the weight of the individual study in the meta-analysis; black diamond, pooled estimate with length depicting the 95% CI)

Parathyroid imaging (^{99m}Tc): AC/AL-CT

Country	n	CTDI$_{vol}$ (mGy) mean	CTDI$_{vol}$ (mGy) 95% CI	CTDI$_{vol}$ (mGy) weight	DLP (mGy.cm) mean	DLP (mGy.cm) 95% CI	DLP (mGy.cm) weight
Bulgaria	4	5.7	[4.6, 6.8]	39.7%	192	[150, 233]	33.5%
Nordic countries	13	4.1	[2.6, 5.6]	24.3%	150	[108, 191]	33.5%
Switzerland	8	4.4	[2.7, 6.1]	20.2%	142	[77, 207]	13.5%
United Kingdom	36	7.4	[2.0, 12.8]	2.2%	164	[89, 239]	10.2%
Australia	-	-	-	-	205	[38, 372]	2.0%
USA	35	4.3	[2.2, 6.4]	13.5%	152	[64, 240]	7.3%
**Pooled estimate**		**4.9**	**[4.1, 5.7]**	**100%**	**166**	**[142, 190]**	**100%**

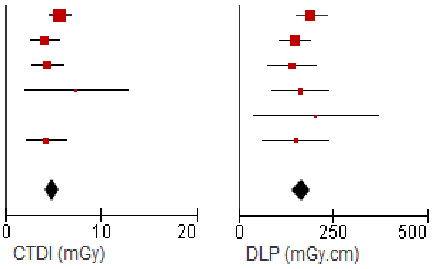

CTDI$_{vol}$:
Heterogeneity: $Tau^2 = 0.13$; $Chi^2 = 4.69$, df = 4 ($P = 0.32$); $I^2 = 15\%$
Test for overall effect: Z = 11.78 ($P < 0.00001$)
DLP:
Heterogeneity: $Tau^2 = 0.00$; $Chi^2 = 2.95$, df = 5 ($P = 0.71$); $I^2 = 0\%$
Test for overall effect: Z = 13.64 ($P < 0.00001$)

Parathyroid imaging (^{99m}Tc): D-CT

Country	n	CTDI$_{vol}$ (mGy) mean	CTDI$_{vol}$ (mGy) 95% CI	CTDI$_{vol}$ (mGy) weight	DLP (mGy.cm) mean	DLP (mGy.cm) 95% CI	DLP (mGy.cm) weight
Nordic countries	1	15.6	[4.8, 26.4]	15.8%	500	[270, 729]	24.3%
Switzerland	5	15.6	[10.2, 21.0]	62.8%	365	[-13, 743]	11.1%
United Kingdom	5	13.4	[4.1, 22.7]	21.4%	287	[216, 358]	64.6%
**Pooled estimate**		**15.1**	**[10.8, 19.4]**	**100%**	**347**	**[211, 484]**	**100%**

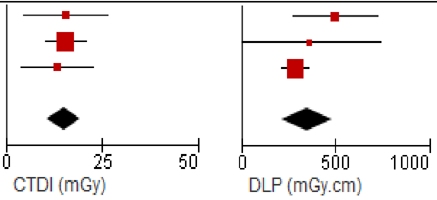

CTDI$_{vol}$:
Heterogeneity: $Tau^2 = 0.00$; $Chi^2 = 0.17$, df = 2 ($P = 0.92$); $I^2 = 0\%$
Test for overall effect: Z = 6.89 ($P < 0.00001$)
DLP:
Heterogeneity: $Tau^2 = 6174.32$; $Chi^2 = 3.12$, df = 2 ($P = 0.21$); $I^2 = 36\%$
Test for overall effect: Z = 5.00 ($P < 0.00001$)

Fig. S6 Forest plot of reported mean CTDI$_{vol}$ and DLP values for ^{99m}Tc SPECT/CT parathyroid imaging divided by studies using an attenuation correction and anatomical localisation CT (AC/AL-CT) and a diagnostic CT (D-CT) (n, number of included SPECT/CT systems; CI, confidence interval; df, degrees of freedom; red square, size proportional to the weight of the individual study in the meta-analysis; black diamond, pooled estimate with length depicting the 95% CI)

Thyroid scan (^{99m}Tc): AC/AL-CT

		CTDI$_{vol}$ (mGy)			DLP (mGy.cm)		
**Country**	**n**	**mean**	**95% CI**	**weight**	**mean**	**95% CI**	**weight**
Bulgaria	4	2.1	[1.6, 2.6]	56.2%	86	[66, 106]	65.8%
Switzerland	8	4.4	[2.7, 6.1]	43.8%	142	[77, 207]	34.2%
**Pooled estimate**		**3.1**	**[0.8, 5.3]**	**100%**	**105**	**[53, 157]**	**100%**

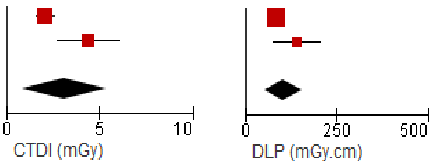

CTDI$_{vol}$:
Heterogeneity: Tau² = 2.25; Chi² = 6.74, df = 1 (P = 0.009); I² = 85%
Test for overall effect: Z = 2.72 (P = 0.006)
DLP:
Heterogeneity: Tau² = 973.50; Chi² = 2.64, df = 1 (P = 0.10); I² = 62%
Test for overall effect: Z = 3.96 (P < 0.0001)

Fig. S7 Forest plot of reported mean CTDI$_{vol}$ and DLP values for ^{99m}Tc SPECT/CT thyroid scan using an attenuation correction and anatomical localisation CT (AC/AL-CT) (n, number of included SPECT/CT systems; CI, confidence interval; df, degrees of freedom; red square, size proportional to the weight of the individual study in the meta-analysis; black diamond, pooled estimate with length depicting the 95% CI)

Post-thyroid ablation (^{131}I): AC/AL-CT

		CTDI$_{vol}$ (mGy)			DLP (mGy.cm)		
**Country**	**n**	**mean**	**95% CI**	**weight**	**mean**	**95% CI**	**weight**
Bulgaria	4	4.1	[1.4, 6.8]	10.3%	120	[83, 157]	45.9%
United Kingdom	15	4.5	[3.5, 5.4]	85.1%	141	[102, 180]	44.6%
Canada	1	6.5	[2.4, 10.5]	4.6%	281	[138, 424]	9.5%
**Pooled estimate**		**4.6**	**[3.6, 5.4]**	**100%**	**145**	**[96, 193]**	**100%**

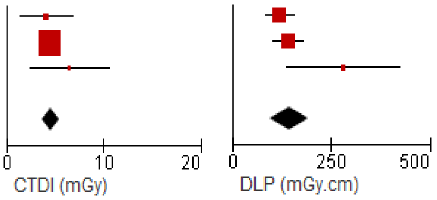

CTDI$_{vol}$:
Heterogeneity: Tau² = 0.00; Chi² = 1.01, df = 2 (P = 0.60); I² = 0%
Test for overall effect: Z = 10.28 (P < 0.00001)
DLP:
Heterogeneity: Tau² = 935.51; Chi² = 4.71, df = 2 (P = 0.09); I² = 58%
Test for overall effect: Z = 5.93 (P < 0.00001)

Fig. S8 Forest plot of reported mean CTDI$_{vol}$ and DLP values for ^{131}I SPECT/CT post-thyroid ablation imaging using an attenuation correction and anatomical localisation CT (AC/AL-CT) (n, number of included SPECT/CT systems; CI, confidence interval; df, degrees of freedom; red square, size proportional to the weight of the individual study in the meta-analysis; black diamond, pooled estimate with length depicting the 95% CI)
